# Reattachment of the Multifidus Tendon in Lumbar Surgery to Decrease Postoperative Back Pain: A Technical Note

**DOI:** 10.7759/cureus.6366

**Published:** 2019-12-12

**Authors:** Neil Klinger, Emre Yilmaz, Dia R Halalmeh, R. Shane Tubbs, Marc D Moisi

**Affiliations:** 1 Neurological Surgery, Wayne State University School of Medicine, Detroit, USA; 2 Surgery, Swedish Neuroscience Institute, Seattle, USA; 3 Neurological Surgery, Detroit Medical Center, Detroit, USA; 4 Clinical Anatomy, Seattle Science Foundation, Seattle, USA

**Keywords:** multifidus muscle, lumbar surgery, back pain, reattachment, postoperative

## Abstract

The posterior midline approach to the lumbar spine requires significant manipulation of the paraspinal muscles. Muscle detachment and retraction results in iatrogenic damage such as crush injury, devascularization, and denervation, all of which have been associated with postoperative pain. The muscle most directly affected by the posterior approach is the lumbar multifidus (LM), the largest and most medial of the deep lumbar paraspinal muscles. The effects of the posterior approach on the integrity of the LM is concerning, as multiple studies have demonstrated that intraoperative injuries sustained by the LM lead to postoperative muscle atrophy and potentially worsening low back pain. Given the inevitability of intraoperative paraspinal muscle manipulation when using the posterior approach, this technical note describes methods by which surgeons may minimize LM tissue disruption and restore the anatomical position of the LM to ultimately expedite recovery, minimize postoperative pain, and improve patient satisfaction.

## Introduction

The standard open posterior midline approach to the lumbar spine is widely used in the treatment of various spinal disorders. However, procedures using the posterior approach are not without challenges and risks. Most commonly employed for decompression and fusion, the traditional posterior approach calls for significant manipulation of the paraspinal muscles, with muscle detachment and retraction that may potentially result in iatrogenic damages such as crush injury, devascularization, and denervation, all of which have been associated with postoperative pain [1-11]. The muscle most directly affected by the posterior approach is the lumbar multifidus (LM) [5]. The largest and most medial of the deep lumbar paraspinal muscles and innervated by the medial branch of the dorsal rami, the LM originates from the sacrum and the ilium and inserts into the spinous processes and laminae of the lumbar vertebrae in a fanning pattern [12]. Upon superficial exposure, the tendon origin of LM is detached from the spinous process and retracted laterally with self-retaining retractors to obtain an adequate surgical window. In cases involving the surgical removal of the spinous processes, the LM is unable to be repaired and returned to its anatomical position. This process places the LM at a significant risk of intraoperative injury that may directly and indirectly lead to negative postoperative outcomes.

The effects of the posterior approach on the integrity of the paraspinal musculature, particularly the LM, is concerning as studies have linked paraspinal muscle integrity to low back pain (LBP). Numerous reports have shown that patients with postoperative LBP exhibit gross and histological signs of LM degeneration, such as muscle atrophy (decreased cross-sectional area [CSA]), LM intramuscular adipose tissue accumulation, and LM denervation [1, 2, 4, 6, 8-10, 13-15]. These findings have been attributed, in part, to the imprecise nature of the LM dissection as well as the intraoperative use of self-retaining retractors, both of which have been shown to result in ischemic necrosis, scar tissue formation, denervation, and atrophy [5, 8, 10, 14]. Given these findings and the inevitability of intraoperative paraspinal muscle manipulation, the following technical report describes methods by which surgeons may minimize tissue disruption and respect the anatomical position of the LM to ultimately expedite recovery, minimize postoperative pain, and improve patient satisfaction.

## Technical report

**Figure 1:**
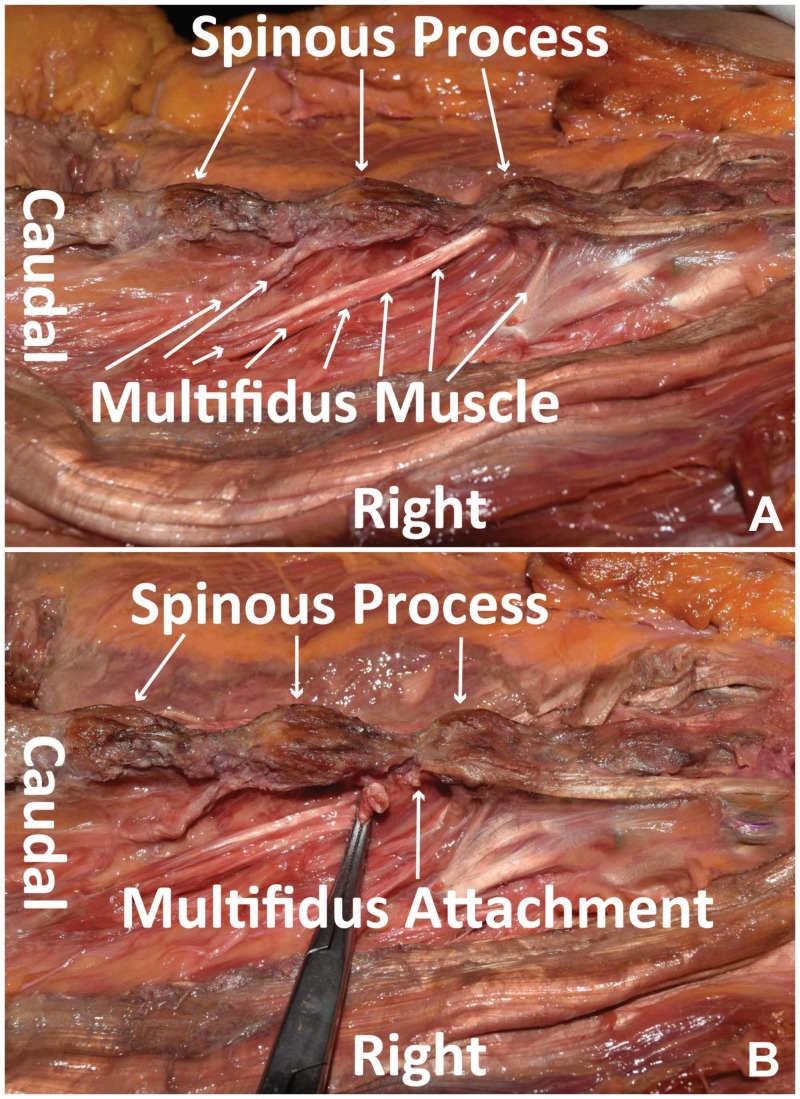
Multifidus muscle identification and detachment (A) Cadaveric dissection using wide retraction to display the LM in natural anatomical position, with the LM inserting rostrally into the spinous processes of the lumbar vertebrae in a fanning pattern (small white arrows). (B) Tendon insertion of the LM sharply divided with a residual cuff on the spinous process. LM, lumbar multifidus

**Figure 2:**
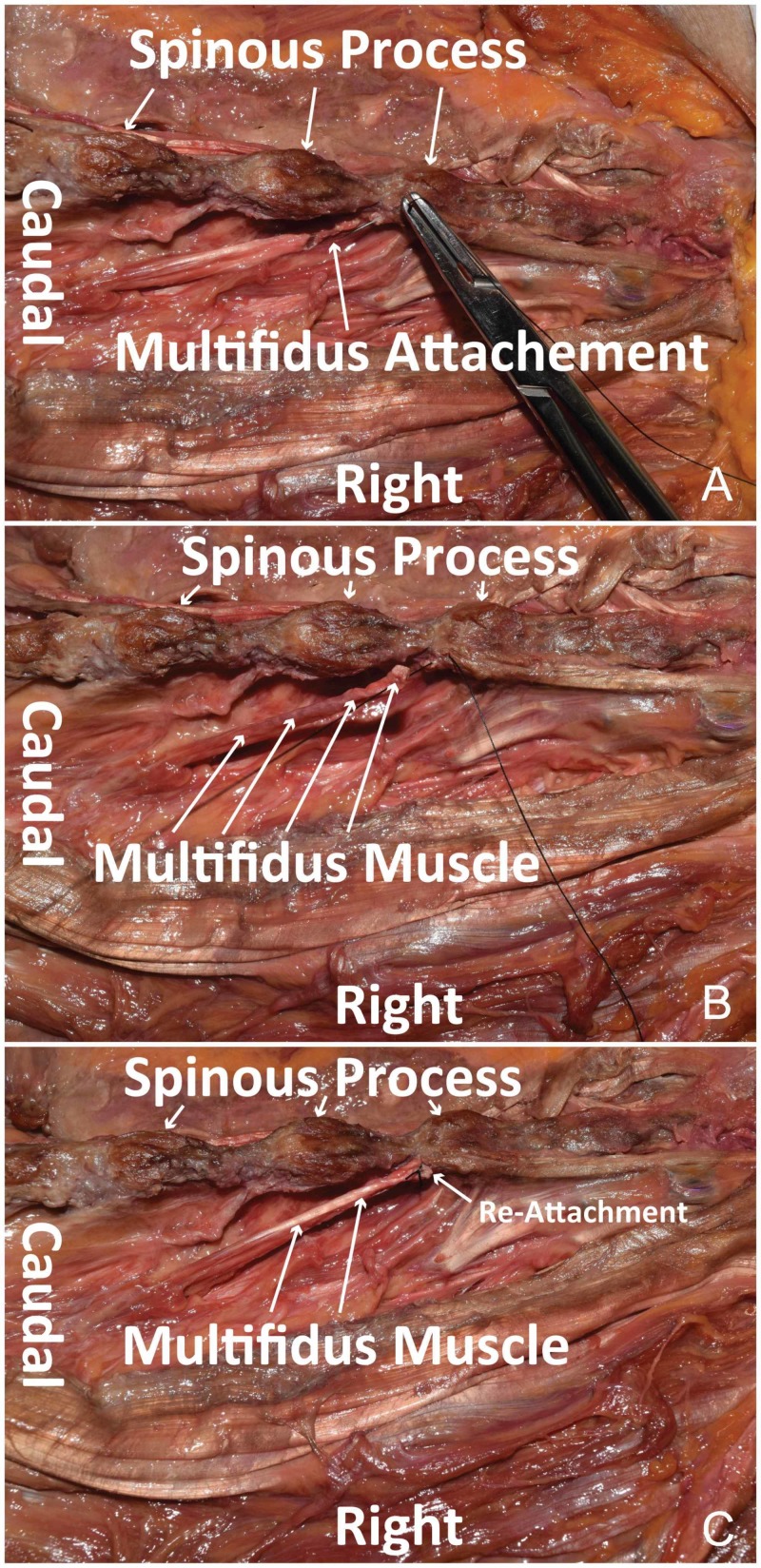
Multifidus muscle reattachment Cadaveric dissection using wide retraction to display reattachment of the LM to the tendon insertion at the spinous process. (A) Suture needle through the residual tendon cuff of the LM attached to the spinous process. (B) Residual tendon cuff of the LM is re-approximated to the body of the LM. (C) Suture placed to reattach the tendon insertion of the LM to the body of the LM. LM, lumbar multifidus

The operation is started in the usual fashion. The initial skin incision is made based on the intraoperative X-ray to identify the target level(s). Midline dissection is continued using Bovie electrocautery down to the level of the spinous process. Once the spinous process is reached, dissection is continued in the paramedian plane 5 mm lateral to the spinous process. The tendon of the multifidus is then identified at its origin on the spinous process (Figure 1A). Care is taken to identify this before proceeding at each level of the planned operation. The tendon is then sharply divided, leaving a small cuff on the spinous process. This will serve as the point of reattachment at the termination of the procedure (Figure 1B). From here, the procedure continues. Once completed, re-approximation of the LM begins by using 2-0 Vicryl suture (Ethicon Inc., Somerville, NJ, USA). The medial end of the free tendon is reattached to the residual cuff on the spinous process (Figure 2) at each level dissected. If a complete laminectomy is performed, the spinous process is completely removed. In these cases, the ends of bilateral tendons are adjoined to each other. Regular closure ensues from here.

## Discussion

The posterior approach to the lumbar spine, most commonly used for decompression and fusion procedures, involves significant manipulation of the paraspinal muscles, particularly the LM [1-6]. Upon standard superficial exposure, the tendon origin of the LM is dissected from the spinous process and, together with other paraspinal muscles, is retracted laterally to obtain an adequate surgical window [6, 14]. Such manipulation places the LM at a significant risk of iatrogenic damage resulting from crush injury, devascularization, and denervation, all of which have been associated with postoperative pain and muscle atrophy [1-4]. Cases involving the surgical removal of the spinous processes further disrupt the LM due to the inability to return the LM to its usual anatomical position.

The effect of the posterior approach on the integrity of the paraspinal musculature is of concern, as LBP has been associated with pathological paraspinal muscle integrity and morphology. Studies on non-surgical patients have shown that patients suffering from LBP have smaller LM CSAs [16], increased intramuscular fat content [17], and histochemical evidence of muscle fiber atrophy with signs of intramuscular fibrosis [18]. These findings are also seen among surgical patients, with multiple studies demonstrating that mechanical, ischemic, and denervating intraoperative injuries sustained by the LM lead to postoperative muscle atrophy [8, 11, 19]. Using MRI to measure paraspinal muscle CSA, a study by Kim et al. found that postoperative open posterior lumbar surgery patients had a significant decrease in LM CSA 21 months after discharge as compared with patients undergoing percutaneous procedures [19]. These findings are complemented by tissue and histology studies that found that LM specimens of open posterior lumbar surgery patients exhibited significant muscle fiber atrophy [7, 20], increased intramuscular fibrosis, and increased intramuscular fatty infiltration [1, 15]. These pathological findings coincide with those seen in non-surgical patients suffering from LBP and serve as a potential explanation for the discouraging incidence of LBP in patients following open posterior procedures of the lumbar spine. Taking these findings into consideration, we have presented methods by which surgeons can attempt to restore the anatomical integrity of the LM in an attempt to optimize recovery and avoid debilitating postoperative LBP.

As described above, our methods of either reattaching the LM to the spinous process or attaching the ends of bilateral LM tendons to each other following laminectomy find their rationale in the successes of minimally invasive surgeries of the lumbar spine. Numerous studies have shown that minimally invasive surgeries, by avoiding the detachment of muscle tendons, decreasing muscle retraction and minimizing the size of the surgical corridor, maintain the integrity of the paraspinal musculature, are less damaging to the LM, and are associated with reduced incidences of postoperative LBP [1, 9, 13, 19]. In a study by Fan et al., patients undergoing minimally invasive procedures of the lumbar spine had less postoperative back pain, LM atrophy, fatty infiltration, and functional disability as compared with patients undergoing conventional open posterior approach [1]. By serially tracking creatinine kinase (CK) levels 1, 3 and 5 days postoperatively, the authors also demonstrate that minimally invasive surgeries resulted in significantly lower CK levels postoperatively compared with open procedures, further suggesting decreased muscle damage [1]. These findings suggest that minimally invasive procedures, by emphasizing the avoidance of soft tissue injury and displacement, are able to decrease the incidence of postoperative LBP and muscular injury in patients.

## Conclusions

Minimally invasive approaches are not always possible in the surgical treatment of spinal pathology. As such, manipulation of the paraspinal musculature is unavoidable. However, steps can be taken intraoperatively to mitigate damage to LM, including careful retraction and dissection as described earlier. We propose the surgical technique of re-approximation and repair of LM as a way to minimize LM damage and restore paraspinal anatomical integrity, which may lead to improved outcomes, decreased postoperative pain, and increased patient satisfaction.
